# Implementation of smoking cessation policy at the antelope house in Southern Health NHS Foudation Trust in Southampton

**DOI:** 10.1192/bjo.2021.287

**Published:** 2021-06-18

**Authors:** Sunday Neru, Edet Ededet

**Affiliations:** Antelope House, Southern Health NHS Foundation Trust, Brintons Terrace, Southampton

## Abstract

**Aims:**

To ensure that health care practitioners at Antelope house, Southern Health NHS Foundation Trust in Southampton are providing service users information, advice and stop smoking support in line with smoke free policies in the trust.

**Background:**

Smoking is the single largest preventable cause of ill health and premature death in England. Cigarette smoking causes a wide range of diseases and medical conditions like cancers, heart diseases and stroke.

The prevalence of stroke is extremely high among people with mental health problems especially those admitted to hospital.

Stopping smoking reduces the risk of developing preventable diseases and premture death.

These are the background behind this audit.

**Method:**

Data were collected using the following ways:

Use of desinged questionaire.

Looking into Rio elecronic records

Standard used and compared against was Southern Health NHS Foundation Trust '' smoke free trust policies'

20 cases were looked into and examined.

**Result:**

Most staff are not implementing the Trust no smoking policies well and documentation of the informtion given are not complete.

Most service users prefer to use e-cigarettes.

Most people between 30 and 50 years old range do not smoke.

For those of clozapine, the impact of cigarettes smoking not explained.

**Conclusion:**

The trust smoke free policies are not well implemented by health care practitioners at Antelope house mental health unit, Southern Health NHS Foundation Trust in Southampton.

